# Immediate Partial Breast and Nipple–Areola Complex Reconstruction Using a Superficial Circumflex Iliac Artery Perforator Flap

**DOI:** 10.1055/a-2223-5325

**Published:** 2024-01-24

**Authors:** Gemma Pons, Lucía Sisternas, Jaume Masià

**Affiliations:** 1 Department of Plastic and Reconstructive Surgery, Hospital de la Santa Creu i Sant Pau, Barcelona, Spain

**Keywords:** nipple-areola complex reconstruction, SCIP flap, partial breast reconstruction

## Abstract

The superficial circumflex iliac artery perforator (SCIP) flap is a versatile flap that has been described for various applications, mostly for lower extremity coverage and head and neck reconstructions. However, there are few publications reporting its use for breast reconstruction, mainly because of its low volume availability. In this article, we present the case of a patient who successfully underwent a partial breast and immediate nipple–areola complex (NAC) reconstruction with an SCIP flap. She had been previously reconstructed with an implant after a nipple-sparing mastectomy, but the NAC turned out to be involved with cancer needing further resection. Our goal with this article, is to introduce a novel concept for addressing partial breast and NAC reconstruction and mostly, to illustrate the importance of an adaptable surgical plan based on every individual case emphasizing the versality of microsurgery for breast cancer reconstruction.

Breast reconstruction is a key element to the successful treatment of breast cancer and it also constitutes one of the main concerns of patients regarding breast surgery sequelae. If we could ask patients which would be their “wishes” in breast reconstruction, the answer would probably be to recover their “real” breast. That means a natural breast, with no scars and no donor site morbidity and everything, if possible, achieved in a single procedure. But unfortunately, this is closer to regenerative surgery than what can be offered nowadays.

When faced with a partial breast reconstruction, it is generally considered to be oncologically safe and appropriate. Building upon this foundation, our primary objective is to offer the most aesthetically pleasing and functionally effective reconstruction possible. Ideally, we aim to accomplish this in a single surgical procedure, taking into careful consideration the patient's preferences and desires throughout the process.

Herein, in this article we present the case of a woman who underwent an immediate partial breast and nipple–areola complex (NAC) reconstruction with a superficial circumflex iliac artery perforator (SCIP) flap. She had a previous breast reconstruction with an implant after a nipple-sparing mastectomy, but there was residual cancer at the NAC, needing further resection of the complex.

Although there are some publications reporting the usefulness of the SCIP flap for autologous breast reconstruction despite its low volume, 1 2 3 4 no article reports the use of the SCIP flap for a partial breast and NAC reconstruction in a previous implant-based breast reconstruction.

Our goal with this article is to introduce a novel concept for addressing partial breast and NAC reconstruction using an SCIP flap and also to emphasize the importance of an individualized plan for each patient with the aim of offering the best reconstructive option.

## Clinical Details

**Fig. 1:**
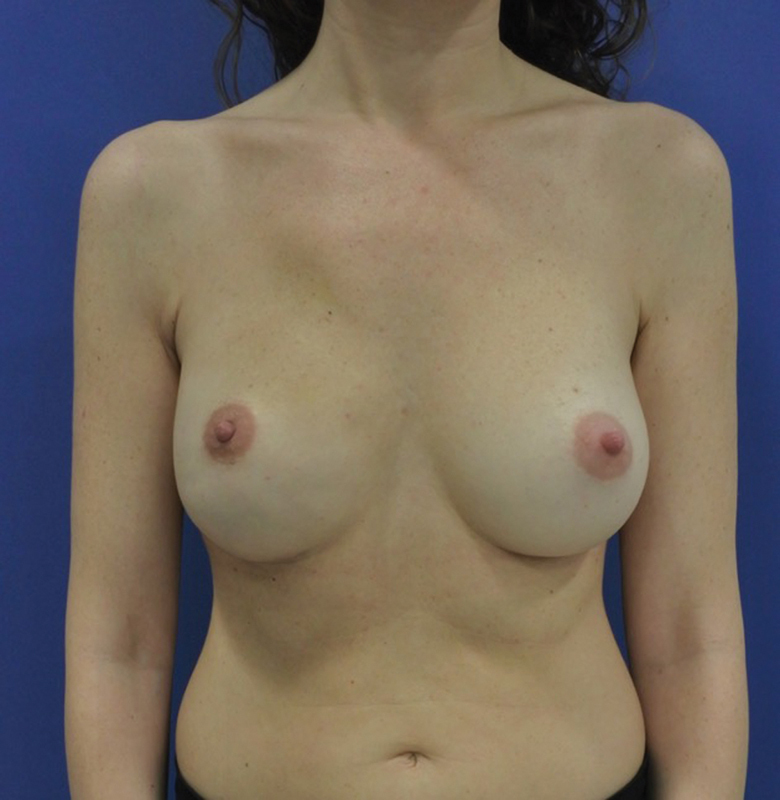
Breast reconstruction with prepectoral implant and acellular dermal matrix with a satisfactory result.

**Fig. 2:**
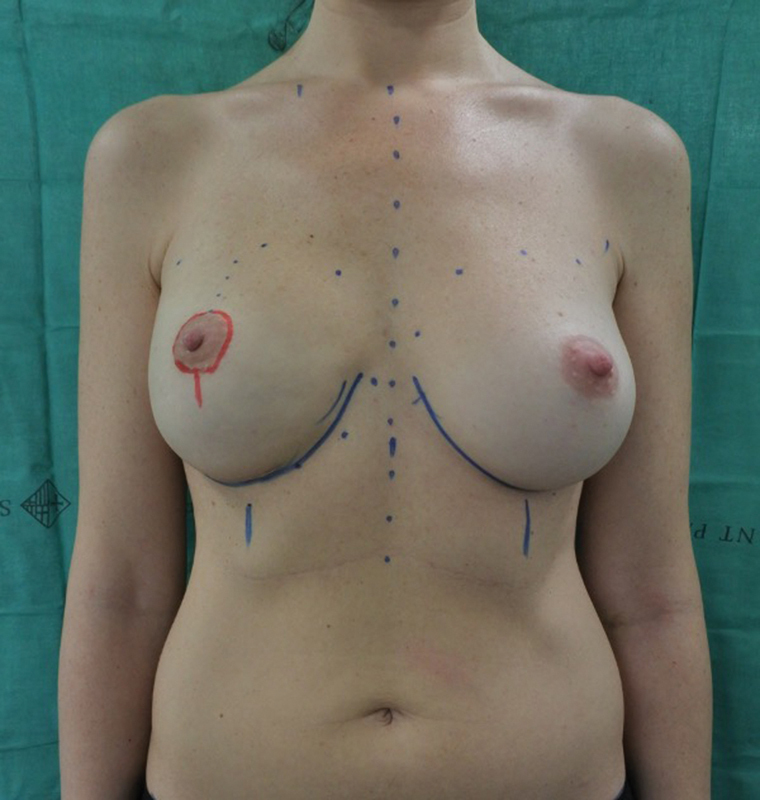
The NAC resection was performed with a periareolar and short midline vertical incision. NAC, nipple–areola complex.

**Fig. 3:**
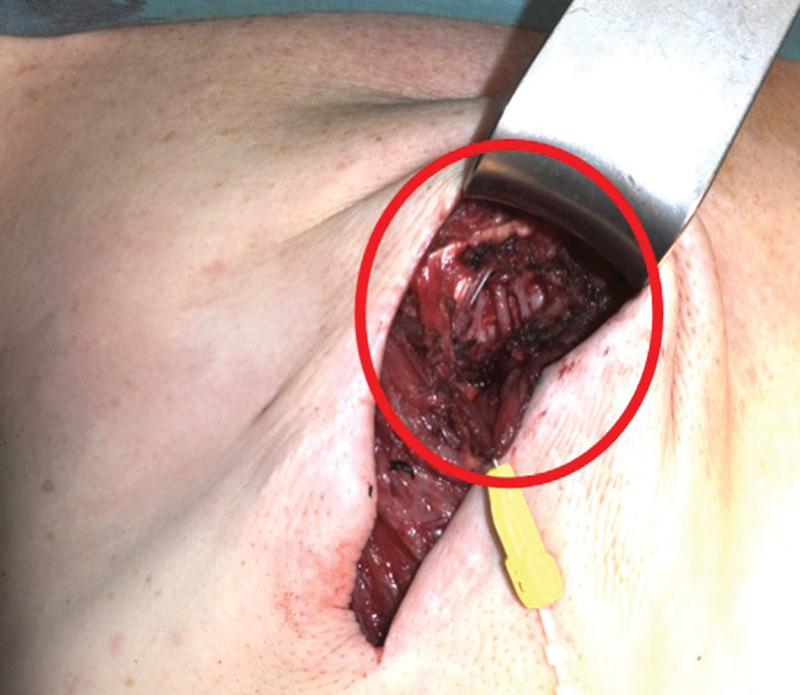
Preparation of the internal mammary vessels as recipient vessels with total rib preservation in the third intercostal space.

**Fig. 4:**
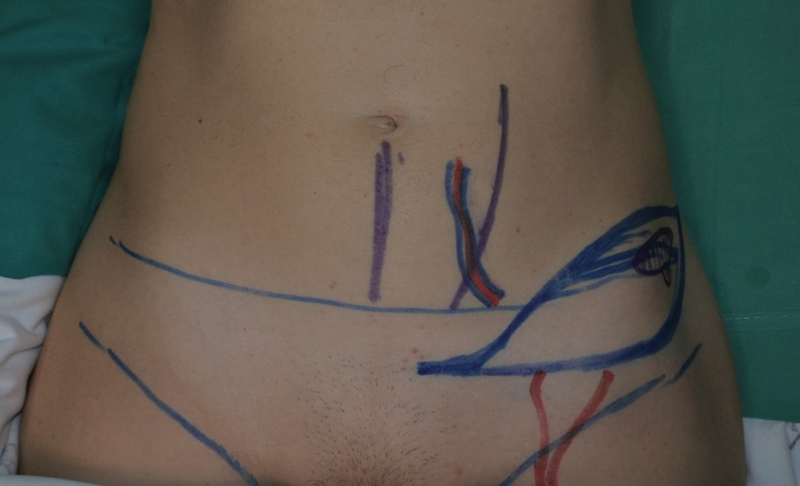
SCIP flap design for NAC reconstruction. NAC, nipple–areola complex; SCIP, superficial circumflex iliac artery perforator.

**Fig. 5:**
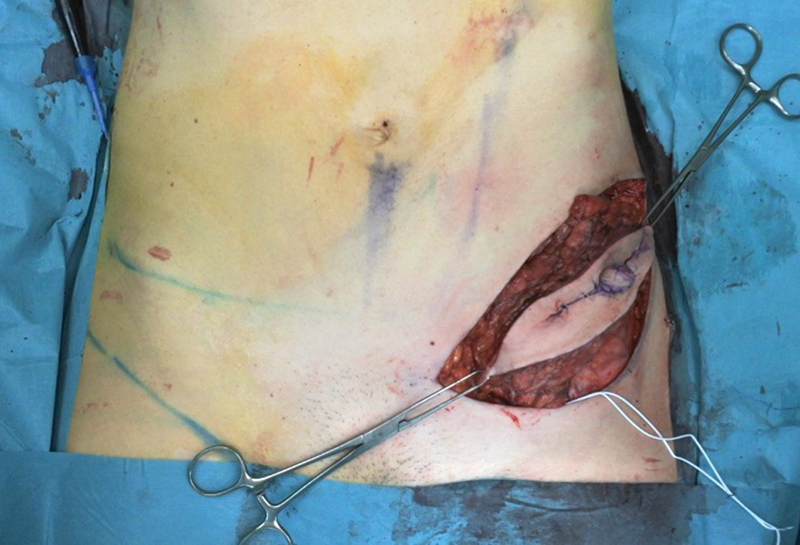
NAC was recreated immediately with a Star-Flap design on the SCIP flap once raised. NAC, nipple–areola complex; SCIP, superficial circumflex iliac artery perforator.

**Fig. 6:**
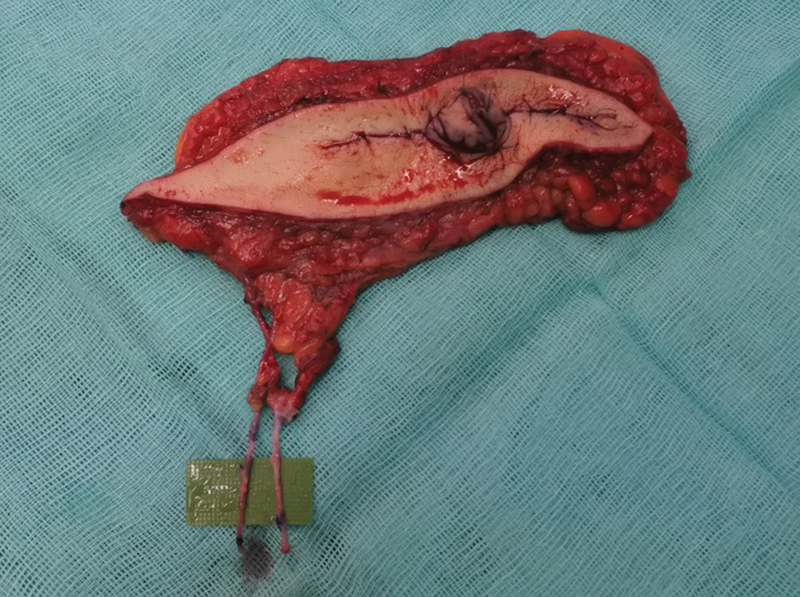
The artery and the vein of the SCIP flap were anastomosed end-to-end to the superficial epigastric vein graft, which was previously divided in two grafts. SCIP, superficial circumflex iliac artery perforator.

**Fig. 7:**
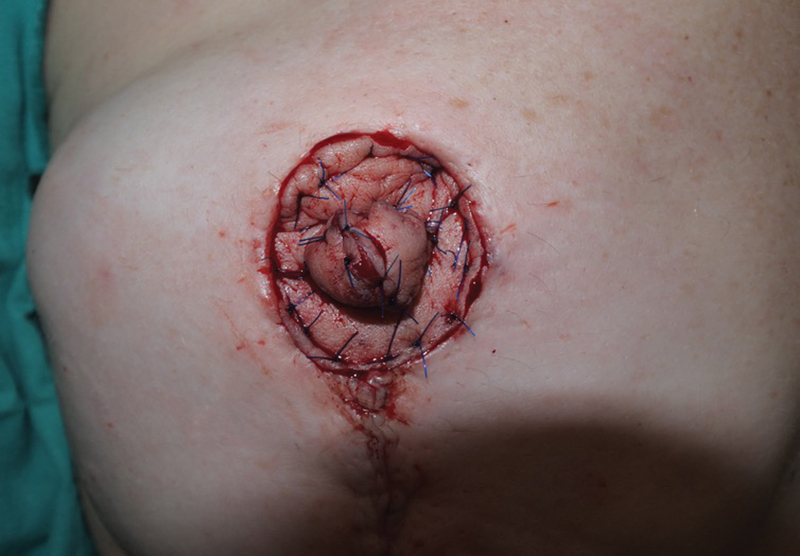
Immediate postoperative result after NAC reconstruction. The flap was shaped and adapted to the defect. NAC, nipple–areola complex.

**Fig. 8:**
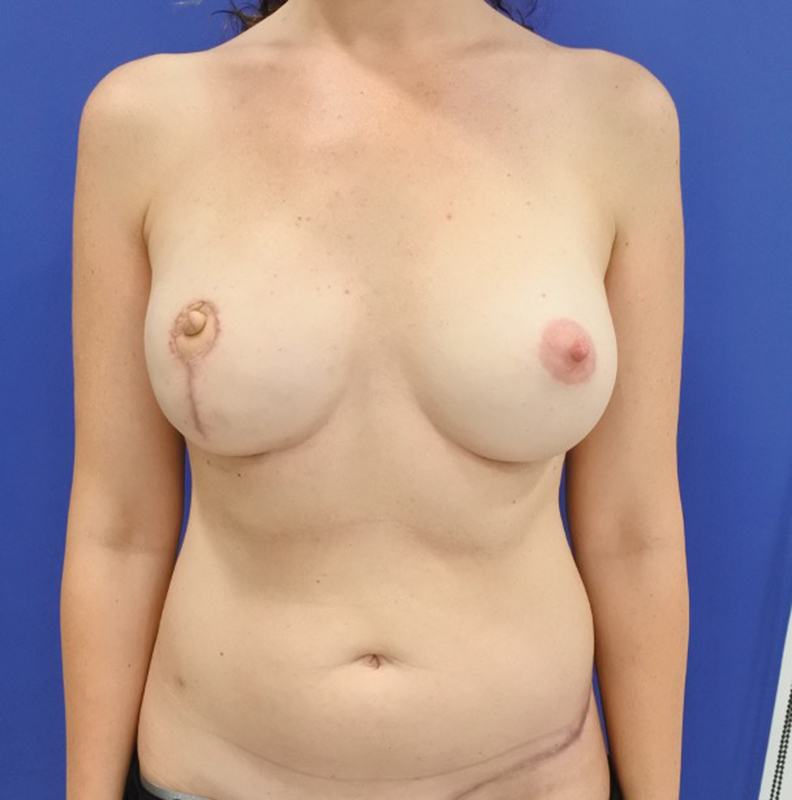
Aesthetically pleasing result with good symmetry of the breasts and natural appearance at 2 months.

**Fig. 9:**
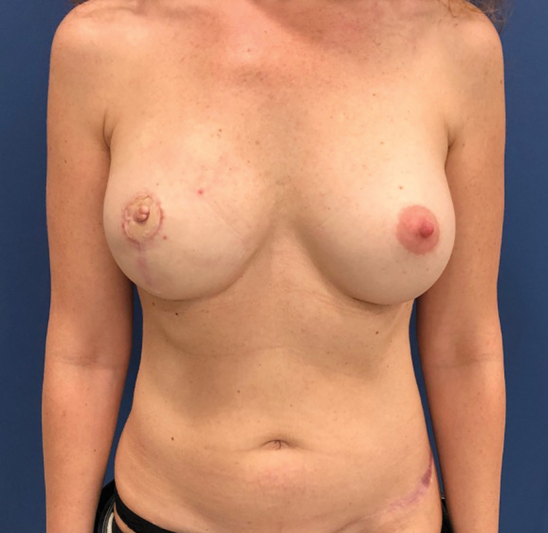
Postoperative result at 6 months.

**Fig. 10:**
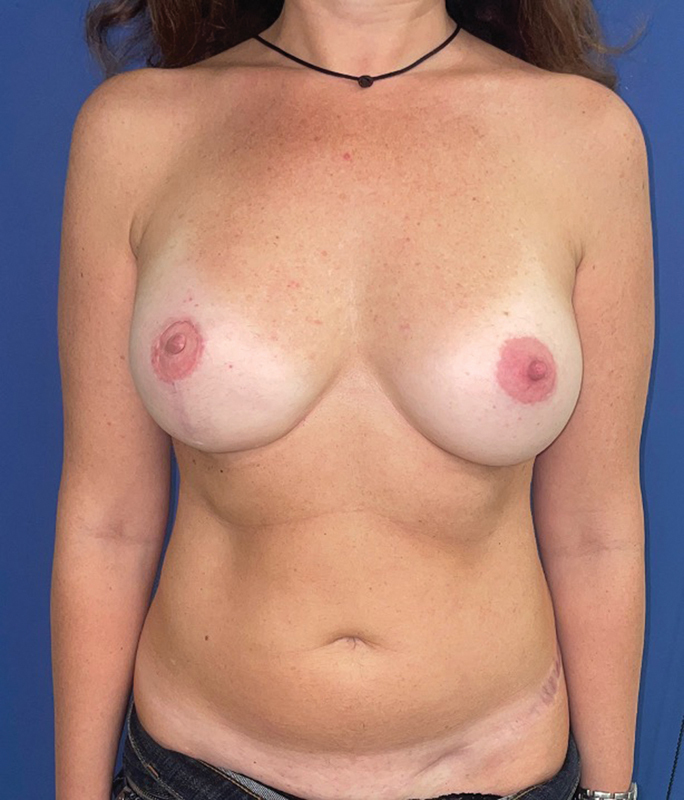
Two years postoperative final result.

**Fig. 11:**
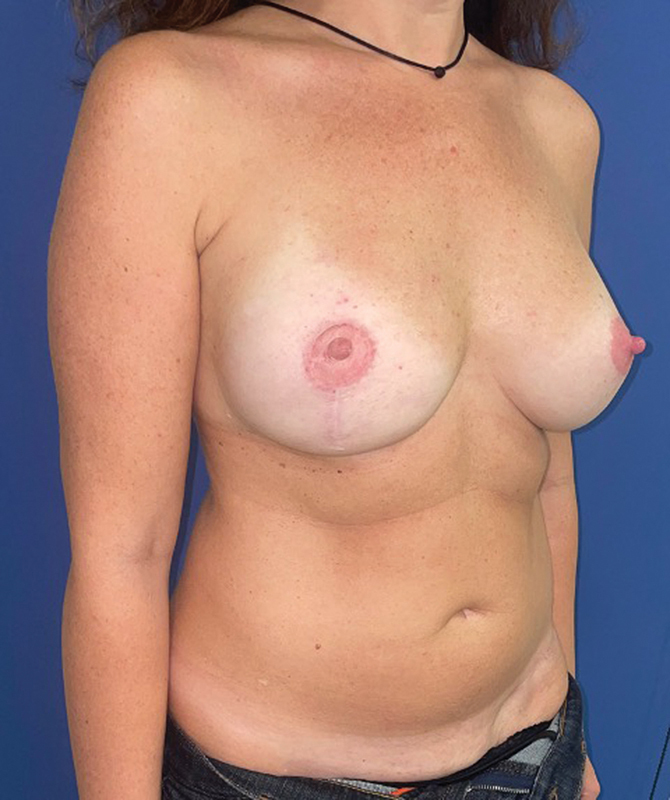
Two years postoperative final result.

**Fig. 12:**
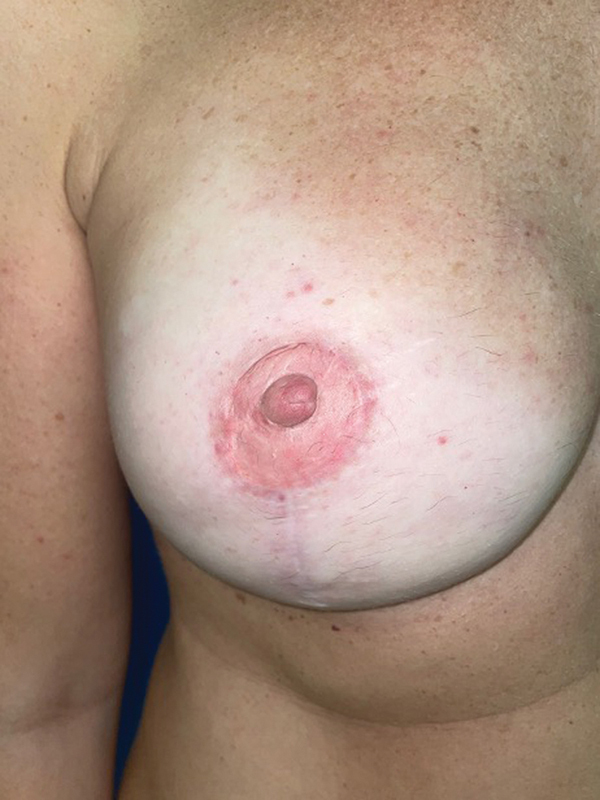
Tattooing of the NAC. NAC, nipple–areola complex.

A 42-year-old patient underwent a nipple-sparing total mastectomy and sentinel lymph node biopsy for right breast cancer at a private center in February 2018. Breast reconstruction with prepectoral implant and acellular dermal matrix was performed with satisfactory result ( Fig. 1 ). A 360-mL implant with anatomic shape and moderate projection was utilized (Allergan N-27-MM130-360). The pathology result revealed positive retroareolar margins and the patient was referred to our center for nipple–areolar complex resection and reconstruction. After a careful physical assessment and with the aim of trying to offer the best for our patient, we agreed to perform an SCIP flap for the immediate NAC reconstruction. Surgery was performed under general anesthesia in a supine position. A periareolar and short midline vertical incision was made including the NAC ( Fig. 2 ). The prepectoral implant was removed, maintaining the already integrated acellular dermal matrix. The incision was used to prepare the internal mammary vessels as recipient vessels with total rib preservation in the third intercostal space ( Fig. 3 ). At the groin region, a line was drawn between the left anterior superior iliac spine and the pubis. With the aid of preoperative Angio-CT and Doppler sonography, the point where the superficial branch of the superficial circumflex iliac artery perforator (SCIP) crosses over the cribriform fascia was marked. A 15 cm (long) × 7 cm (wide) skin paddle was designed over this line including the perforator ( Fig. 4 ). First, a skin incision was made over the site where the main pedicle (superficial branch of SCIA) lay, on the medial and lower part of the skin paddle. The dissection proceeded from distal to proximal and lateral to medial, allowing incorporation of the superficial branch of the SCIA with an axial pattern. 5 Once the main pedicle was located and isolated with the flap, the lower incision was performed. Under direct vision, the pedicle was dissected to its origin at the femoral vessels. The superficial inferior epigastric vein was dissected 6 cm to be used as a graft to lengthen both the artery and the vein of the pedicle. The final pedicle with the vein graft measured 10 cm. Donor site was closed directly. The NAC was recreated immediately with a Star-Flap design in the SCIP flap ( Fig. 5 ). On a separate table and while the donor site was being closed, the artery and the vein of the SCIP flap were end-to-end anastomosed to the vein graft, which was previously divided in two grafts ( Fig. 6 ). Finally, the SCIP flap was end-to-end anastomosed to the internal mammary vessels. The flap was shaped and adapted to the defect ( Fig. 7 ). Finally, a 400-mL expander was inserted into the breast pocket taking care to avoid pedicle compression. Intraoperative filling was 120 mL. The surgery lasted for 4 hours and 35 minutes. At 2 months postsurgery ( Fig. 8 ), the patient underwent an exchange of the expander (filled with 360 mL) for an implant (360 mL) and also autologous fat grafting to optimize the shape of the flap. She did not undergo radiotherapy ( Fig. 9 ). Assessment of outcome at 2-year follow-up showed an aesthetically pleasing result with good symmetry of the breasts, natural appearance and no donor site complications ( Figs. 10 and 11 ). The patient underwent tattooing of the NAC and was very pleased with functional and aesthetic results ( Fig. 12 ).

## Discussion

The SCIP flap is a versatile flap that has been described for various applications. It provides a very thin, pliable, moderate-size skin paddle which is very useful for reconstructing cutaneous defects. 6 This is the reason why it has been widely used to reconstruct defects over the dorsum of the foot or around the toes, hand and upper limb, and head and neck defects which do not tolerate bulkiness. 7 And because of its low volume availability, it is not widely used for total breast reconstruction.

The evolution of microsurgery in the last 30 years, especially with perforator flaps, has allowed us to create a natural beautiful breast that can mimic the real one. This achievement is founded upon the “like-to-like” principle and tissue specificity. The versatility of microsurgery offers us several reconstructive options that can be adapted to every patient according to their oncologic condition and anatomical constitution. It is very important to carefully select the donor site so that we can offer an extra benefit and minimize the donor site sequalae. All this decision-making process must be done in consensus with the patient body anatomy, so that we can make the right surgical indication individualized for each patient.

The SCIP flap has been previously described as a free flap for breast augmentation, partial and total autologous breast reconstruction. 1 2 3 4 However, there are no articles reporting its use for an NAC reconstruction.

When the patient first came to our center, she had already undergone immediate prepectoral implant breast reconstruction with acelullar dermal matrix on the right breast and a contralateral breast augmentation with an aesthetically pleasant result. After assessing the patient's needs and after a careful exploration, our aim was to perform exclusively the NAC resection and immediate reconstruction, preserving whenever possible the optimal shape and breast pocket that she already had. Therefore, we needed a thin and pliable flap to give us the chance to reconstruct the nipple at the same time, and all of that achieved with the minimal possible morbidity for the patient. We considered the SCIP flap the best indication for our patient, because it offered a relatively low thickness, good pliability, and the donor site scar could be easily hidden. The main disadvantage was that it was technically demanding specially because the artery could show small caliber and the pedicle was short. To overcome these disadvantages, we dissected both the artery and the vein up to the femoral vessels' junction, and we dissected the superficial epigastric vein as a graft to double the pedicle length and reach the internal mammary vessels for the anastomosis at the recipient site and also to reduce the caliber discrepancy. We also had to handle the pedicle with caution to avoid traction or compression when we implanted the expander. Other disadvantages of this technique are the vertical scar on the breast (to access the recipient vessels) and the scar in the groin, which is generally well-concealed. In this case, this alternative indication of the SCIP flap resulted in an aesthetically satisfactory result with good symmetry of the breasts, natural appearance and no donor site complications. In addition, it should also be highlighted that with the use of the SCIP flap, most of the abdominal tissue could be preserved for a deep inferior epigastric artery perforator flap reconstruction, in case that the patient decided to get rid of the implants and undergo a total autologous breast reconstruction in the future.

One perforator flap that is classically used in combination with implants is the thoracodorsal artery perforator flap, 8 9 since it provides a thick fat-subcutaneous tissue in the majority of patients and it spares the muscle. However, it is mainly indicated for partial or total breast reconstruction to provide volume and/or skin to the breast. Related to the case we present, we discarded the thoracodorsal artery perforator (TDAP) flap because the tissue that it provides is not as suitable as the one offered by the SCIP flap. The TDAP flap has more fatty tissue, thick-nonpliable skin, and used as a local flap does not result as idoneous as the SCIP flap because it disrupts the lateral groove of the breast and the skin of the back does not match with the areolar skin.

Another possible alternative to our approach could be to perform the NAC resection, direct closure of the defect and implant exchange for an expander with later reconstruction of the NAC. We discarded this procedure because, although it would avoid the vertical scar on the breast and in the groin, it would result in a horizontal scar on the anterior breast pocket. Such scar is very stigmatic for breast cancer patients and mostly, it decentralizes deprojection of the NAC and the conical shape of the breast, which is almost impossible to restore afterwards. Also, this procedure results in a lack of the skin pocket with high chances of breast shape deformation and probably losing symmetry in relation to the contralateral breast.

About the subject of using an expander instead of a one-staged SCIP flap plus implant, our decision was rooted in our commitment to minimize any potential risk of undue pressure or compression on the pedicle, particularly when utilizing an implant of the same size.

In conclusion, with this article we describe a novel concept for addressing immediate partial breast and NAC reconstruction using an SCIP flap with good functional and aesthetic results. Our attempt to offer the best for our patient, minimizing the mastectomy sequelae and donor site morbidity, drove us to develop this selected procedure. Finally, we would also like to emphasize the importance of an individualized plan searching for the best reconstructive option for our patients considering their anatomical characteristics and desires, whenever possible.
